# Integration of maternal nutrition and pasture management: implications for the performance and parasite resistance of Texel lambs

**DOI:** 10.1007/s11250-026-05325-x

**Published:** 2026-09-04

**Authors:** Thais Fernanda Farias de Souza Arco, Camila Celeste Brandão Ferreira Ítavo, Gelson dos Santos Difante, Vanessa Zirondi Longhini, Fernando de Almeida Borges, Gleice Kelli Ayardes de Melo, Dyego Gonçalves Lino Borges, Priscila Bernardo de Andrade, Luís Carlos Vinhas Ítavo

**Affiliations:** 1 College of Veterinary Medicine and Animal Science of the Federal University of Mato Grosso do Sul, Av. Senador Filinto Muller, 2443, Vila Ipiranga, Campo Grande, MS 79070- 900 Brazil; 2 Federal University of Mato Grosso do Sul, Av. Pedro Pedrossian, 725, Universitário, Paranaíba, MS 79500-000 Brazil; 3 College of Veterinary Medicine and Animal Science of the Federal, University of Mato, Grosso do Sul. Avenue Senador Filinto Muller, 2443. Vila Ipiranga, Campo Grande, MS 79074-460 Brazil

**Keywords:** Maternal nutrition, Pasture management, Marandu grass, Texel lambs, Parasite resistance

## Abstract

Lactating ewes exhibit markedly increased nutritional requirements, and inadequate nutrient supply may impair lactogenesis, leading to reduced milk yield, restricted lamb growth, and substantial losses in productive efficiency. We aimed to evaluate the effects of ewe supplementation at 0.5 and 1.0% BW on the productive performance and gastrointestinal parasitism of lambs maintained on mowed and unmowed *Urochloa brizantha* cv. Marandu pastures. Fifty Texel lambs were allocated with their dams to eight paddocks, four subjected to mowing and four maintained without mowing. Lambs born to ewes supplemented at 1.0% BW exhibited greater performance and body condition score (BCS) at 60 days of age. Advancing lamb age resulted in absolute increases in pasture intake, parasitic burden, and body weight, accompanied by a reduction in BCS (*p* < 0.05). Lambs from ewes receiving 1.0% supplementation showed higher absolute pasture intake between 30 and 60 days of age (*p* < 0.05) and greater parasitic burden at 90 days when maintained on unmowed pastures (*p* < 0.05). Higher NDF and ADF concentrations were observed in the leaf blades of mowed pasture (*p* < 0.05), without compromising lamb productive performance. Supplementation of suckling lambs under a creep-feeding system enhanced resistance and tolerance to gastrointestinal parasitism on pasture, irrespective of pasture management. Moreover, the progressive increase in supplement intake with advancing lamb age contributed to greater growth throughout the 90 days, allowing lambs to be weaned at approximately 30 kg BW, a level considered adequate for initiating the finishing phase and shortening the lamb meat production cycle under tropical pasture conditions.

## Introduction

During the post-partum period, to ewes’ lactating demands increase the nutritional demands, and the inadequate nutrient supply may impair lactogenesis, thereby reducing milk yield, limiting lamb growth, and contributing to substantial productivity losses at the system level (Pesántez-Pacheco et al. 2019). Beyond nutritional constraints, health-related challenges exert a critical influence on production efficiency, with gastrointestinal parasitism being widely recognised as the predominant pathological constraint in small ruminant systems (Coop and Kyriazakis 2001). Suckling lambs are particularly vulnerable, as they demonstrate reduced immunological competence against helminth infection (Melo et al. 2017). This susceptibility varies across physiological categories and genetic backgrounds, with wool breeds exhibiting a higher predisposition to gastrointestinal parasitism compared with hair sheep breeds (Moreira et al. 2021).

However, the implementation of targeted nutritional and management strategies to enhance productive efficiency and mitigate parasitic infection has demonstrated effectiveness in enabling wool sheep production under tropical conditions. Evidence from previous studies indicates that protein–energy supplementation represents a key approach for reducing gastrointestinal nematode burden in suckling and finishing lambs grazing Urochloa pastures (Melo et al. 2017; Silva et al. 2022). In addition, such supplementation has been associated with marked improvements in growth performance and feed utilisation efficiency (Silva et al. 2022).

Lactating ewes typically present higher egg per gram of feces (EPG) of feces pasture contamination (Moreira et al. 2021), which subsequently elevates the risk of parasitic infection in lambs. Despite the relevance of this interaction, there remains a lack of studies evaluating the relationship between productive performance and gastrointestinal nematode infection in suckling lambs as a function of maternal nutritional status during lactation, particularly under tropical conditions, where Urochloa pastures constitute the predominant forage base for livestock systems (Euclides et al. 2019). Notably *Urochloa brizantha* cv. Marandu pastures have been associated with a higher incidence of gastrointestinal nematode infection in lambs compared with other cultivars of the genus (Roberto et al. 2020).

Therefore, the adoption of pasture management strategies capable of mitigating parasitic pressure is essential (Arco et al. 2024), particularly those that modify canopy structure to increase sunlight penetration, thereby reducing gastrointestinal nematode larval development (Roberto et al. 2020). Among these strategies, mechanical pasture height reduction has shown potential relevance.

Considering the high susceptibility of Texel sheep to gastrointestinal parasitism (Bishop et al. 2004) and the favourable microclimatic conditions created by marandu grass (*Urochloa brizantha* cv. Marandu), characterised by high tiller density and a prostrate growth habit conducive to larval survival (Roberto et al. 2020), this study aimed to evaluate the effect of two maternal nutritional levels during lactation (0.5 and 1.0% of body weight) on productive performance, and gastrointestinal nematode infection in Texel lambs grazing mowed and non-mowed marandu pastures. It was hypothesised that increased nutrient intake for lactating ewes, combined with mowed pastures, would enhance productive performance and reduce parasitic infection in lambs.

## Materials and methods

### Location and experimental period

**Fig. 1:**
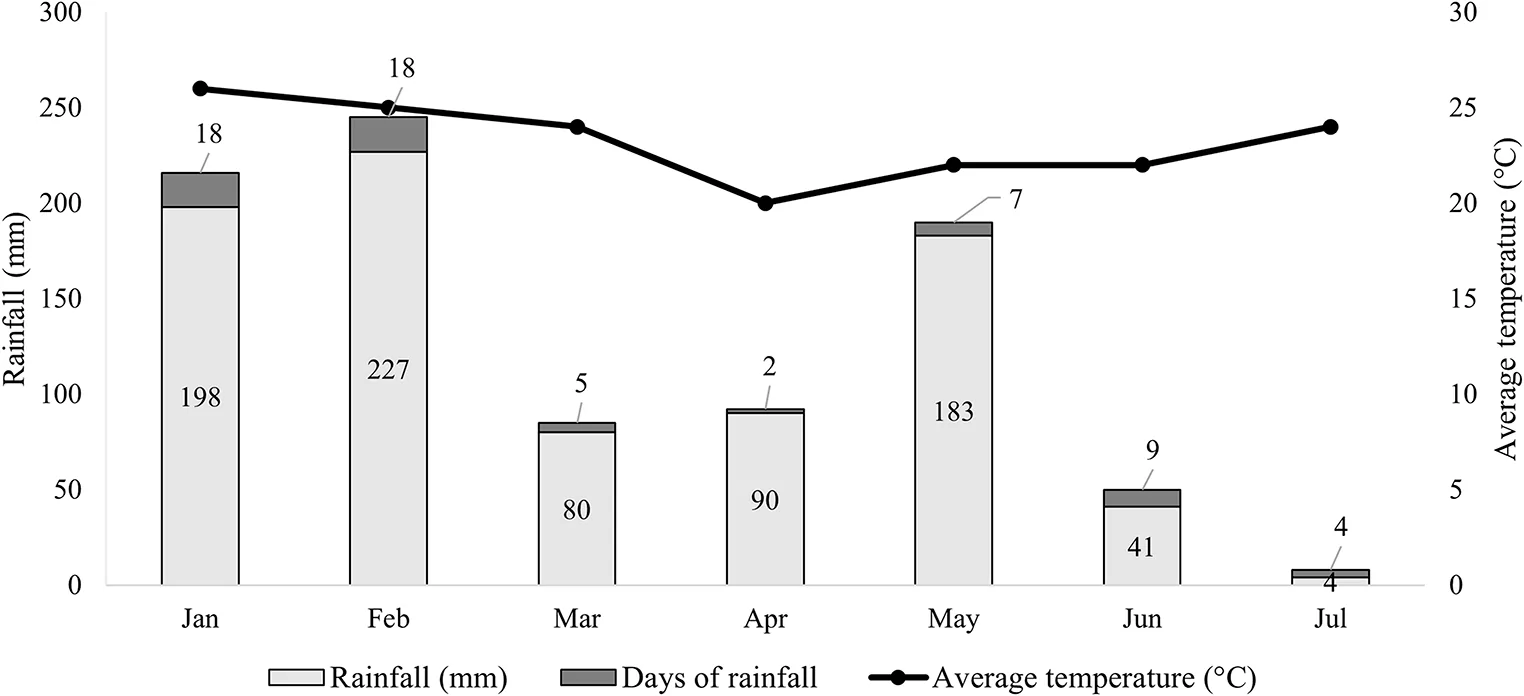
Meteorological data on avarage temperature, rainfall, and number of rainy days from January to July 2020. From January to March (Pre-experimental period); from April to July (Experimental period). INMET, CEMTEC-MS and Agraer (2020)

The experiment was carried out at the Sheep Farming Sector of the School Farm of the Faculty of Veterinary Medicine and Animal Science (FAMEZ), Federal University of Mato Grosso do Sul (UFMS), located in Terenos, Mato Grosso do Sul, Brazil (20°26′34.31″ S; 54°50′27.86″ W; altitude 530.7 m). The assessments were conducted over two distinct periods: The pre-experimental period corresponded to the pasture management phase, during which eight experimentais paddocks were evaluated, including four mowed and four non-mowed paddocks, all of which were rested for 90 days. The experimental period lasted from April to July 2020 and ended with lamb weaning at 90 days of age, corresponding to the suckling phase. Meteorological data were obtained during both the pre-experimental and experimental periods (Fig. 1). These data were provided by the National Institute of Meteorology (INMET, 2020), the Center for Climate and Water Resources Monitoring of Mato Grosso do Sul (Cemtec-MS), and the Agency for Agricultural Development and Rural Extension (Agraer). All procedures were approved by the UFMS Ethics Committee on Animal Use (Protocol No. 862/2017).

### Animals and experimental area

Fifty Texel lambs with an average birth weight of 3.8 ± 1.0 kg were evaluated. The lambs originated from 38 ewes with an average lambing weight of 62.97 ± 7.0 kg and an average body condition score (BCS) of 2.5. The ewes and their lambs were managed in a total grazing area of 3.43 ha, subdivided into eight (8) paddocks of approximately 0.43 ha each, providing two replicates per treatment group. The paddocks, established with *Urochloa brizantha* cv. Marandu, were fenced with smooth wire and sheep-specific netting and were equipped with feeders, water troughs, and creep-feeding facilities accessible only to lambs.

In January, four of the paddocks were mechanically mowed to standardize pasture height at 2 cm above ground level. This management practice aimed to increase soil exposure to solar radiation and improve air circulation, thereby reducing nematode larval survival and promoting faster regrowth with a higher leaf proportion available to lambs during the suckling phase. The pasture mowing management consisted of mechanically lowering the pasture height using 80 hp agricultural tractors. The management of the four paddocks was carried out in January and was not performed again in the respective evaluation months. The four remaining paddoks were not subjected to mowing management. After mowing, all eight paddocks (four mowed and four non-mowed) were rested for 90 days.

The experiment began in April, starting with the first recorded births, and ended in July with the weaning of the lambs, characterizing the suckling phase as an experimental period.

Pasture sampling to determine forage mass on a dry matter (DM) basis was conducted from April to July, corresponding to the experimental evaluation period. Samples were collected at 28-day intervals. In each paddock, six samples were collected using a 0.5 m² metal quadrant (1.0 m × 0.5 m), randomly positioned at representative points within the area. The collected material was weighed and manually separated into leaf blades, stems (stem + sheath), senescent material, and inflorescences.

### Experimental design and diet

At the beginning of the lambing season, the ewes and their lambs were allocated to the eight experimental paddocks. Analyses of lamb productive performance, BCS, and parasitological responses were adjusted using the ewes’EPG at lambing as a covariate, which averaged 1906 ± 1638 EPG. The ewes and lambs were assigned to treatments according to two nutritional levels and two pasture management systems.

The nutritional levels consisted of protein–energy supplementation supplied at 0.5 or 1.0% of the body weight (BW) of lactating ewes kept with their lambs in mowed and non-mowed pastures. Supplementation was offered once daily at 08:00 h. The supplement intended for lambs was provided through a creep-feeding system with free access available 24 h per day. The thirty-eight ewes and their 50 lambs were allocated to two nutritional levels (0.5 and 1.0% of live weight) and two pasture management systems (mowed and non-mowed). The study was conducted in randomized blocks, with treatments distributed in a 2 × 2 factorial design (Table 1). Thus, there were two paddocks (replicates) per treatment, totaling approximately 0.86 ha per experimental trial, distributed as follows: (1) Supplementation of 0.5% of BW + mowed pasture; (2) Supplementation of 0.5% of BW + non-mowed pasture; (3) Supplementation of 1.0% of BW + mowed pasture; and (4) Supplementation of 1.0% of BW + non-mowed pasture. The animals remained on continuous grazing, and paddocks were changed every 28 days among the experimental trials.

**Table 1:** Distribution of ewes and lambs in nutritional treatments (0.5 and 1.0% of body weight) and in grass pastures mowed pasture (MP) and non-mowed pasture (NMP)

	Treatments				
Category^A^	0.5% BW		1.0% BW		Total
	MP	NMP	MP	NMP	
Ewes	10	9	9	10	38
Lambs	13	12	13	12	50
Total	23	21	22	22	88

^A^Treatments 0.5% BW and MP = seven ewes had single births and three ewes had twin births; treatments 0.5% BW and NMP = six ewes had single births and three ewes had twin births; treatments 1.0 BW and MP = five ewes had single births and four ewes had twin births; treatments 1.0 BW and NM = eight ewes had single births and two ewes had twin births

Throughout the suckling phase, the troughs remained installed inside wooden enclosures equipped with access openings measuring 30 cm in height and 20 cm in width, following the recommendations of Melo et al. (2017). The total creep-feeding area measured 1.96 m × 1.30 m.

Daily records of the amounts of protein–energy supplement offered and orts were used to calculate total average daily intake (ADI), as well as ADI at 30, 60, and 90 days of age. The chemical composition of the supplements provided to ewes and lambs, along with supplement intake and the intake of dry matter (DM) and crude protein (CP) for each nutritional level, are presented in Table 2. The total digestible nutrient (TDN) content of the supplements was estimated according to Cappelle et al. (2001) using the equation: TDN (g/kg) = 910.246–5.71588 × NDF.

**Table 2:** Chemical composition of the supplement offered to ewes and lambs during the experimental period

Ewe supplement	
Dry matter (g/kg)	934.9
Mineral matter (g/kg DM)	55.5
Organic matter (g/kg DM)	944.5
Crude Protein (g/kg DM)	214.0
Ether extract (g/kg DM)	97.2
Neutral detergent fiber (g/kg DM)	20.0
Acid detergent fiber (g/kg DM)	8.1
Total digestible nutrients (g/kg DM)^a^	795.7
Lamb supplement	
Dry matter (g/kg)	956.1
Mineral matter (g/kg DM)	47.6
Organic matter (g/kg DM)	952.4
Crude Protein (g/kg DM)	259.9
Ether extract (g/kg DM)	42.8
Neutral detergent fiber (g/kg DM)	18.2
Acid detergent fiber (g/kg DM)	7.2
Total digestible nutrients (g/kg DM)^a^	806.3

^a^Estimated value using the equation by Cappelle et al. (2001): TDN(g/kg) = 910.246–5.71588.NDF

### Growth performance and gastrointestinal parasitism

The productive performance of lambs was assessed at 30, 60, and 90 days of age through body weight measurement and BCS, as well as by calculating average daily gain (ADG) and total weight gain (TWG). ADG was calculated as the difference between weaning and birth weights divided by the number of days in the experimental period (90 days), whereas TWG corresponded to the absolute difference between weaning and birth weights. BCS followed the method described by Russel et al. (1969) using a 1-to-5 scale, in which 1 represents very thin animals and 5 represents obese animals.

Parasitological monitoring was performed every 30 days by collecting individual fecal samples directly from the rectum of lambs to quantify EPG, according to the modified method of Gordon and Whitlock (1939), with a detection sensitivity of 1:25. Lambs were dewormed whenever EPG values reached or exceeded 250, following the recommendations of van Wyk et al. (2006) and Amarante (2009). A combined anthelmintic treatment was administered using albendazole (Valbazen^®^; 5 mg/kg BW, equivalent to 1 mL/20 kg BW), trichlorfon (Triclorsil^®^; 100 mg/kg BW, equivalent to 1 mL/kg BW, diluted at 10 g in 100 mL of water), moxidectin (Cydectin^®^; 0,2 mg/ kg BW, equivalent to 1 mL/kg BW), and eprinomectin (Eprecis^®^; 0.2 mg/kg BW, equivalent to 1 mL/100 kg BW). The adoption of this protocol was based on previously identified phenotypic evidence of multidrug anthelmintic resistance. Gastrointestinal parasitism was assessed using EPG and the proportion of lambs requiring anthelmintic treatment during the intervals of 0–30, 30–60, and 60–90 days’ experimental periods. Fecal samples from the ewes were collected every 30 days to determine EPG. Ewes with EPG values ≥ 500 as described by Arco et al. (2024).

### Analysis of the chemical and morphological composition of the pasture

Samples of the pasture morphological components (leaf blades, stems, senescent material, and inflorescences) were dried in a forced-air oven at 55 °C for 72 h and subsequently ground to pass through a 1-mm screen. Dry matter (DM) content was determined by oven drying at 105 °C for 4 h, following AOAC method 930.15 (2000). Crude protein (CP) was analyzed according to AOAC method 976.05 (2000). Ether extract (EE) was determined using a Tecnal TE-044/1 extractor, following AOAC method 920.39 (2000). Ash concentration was determined by incineration at 600 °C for 2 h in a muffle furnace, according to AOAC method 942.05 (2000). Organic matter (OM) content was calculated as the difference between 100 and the ash percentage.

Neutral detergent fiber (NDF) and acid detergent fiber (ADF) were determined according to the methodology described by Goering and Van Soest (1970), following the INCT–CA F-002/1 and INCT–CA F-004/1 analytical procedures. Total digestible nutrients (TDN) were estimated using the equation proposed by Cappelle (2001): TDN (%) = 83.79 − 0.4171 × NDF.

Daily pasture intake by lambs during the periods 0–30, 30–60, and 60–90 days was estimated using the equation proposed by Costa et al. (2023): Pasture intake (g/day) = 77 × live weight.

### Statistical analyses

The results for productive performance BCS were analyzed by ANOVA using the following statistical model: Yi = µ + Ts + Tp + I + B + (Ts × Tp) + εi, where *Yi* is the observed value for body weight at 30, 60, and 90 days, total weight gain, average daily gain, and BCS at 30, 60, and 90 days; *µ* is the overall mean; *Ts* is the effect of supplementation level (0.5 or 1.0% BW); *Tp* is the effect of pasture management (mowed and non-mowed pasture); *I* is the effect of evaluation period; *B* is the covariate (ewe fecal egg count at lambing); *Ts × Tp* represents the interaction between supplementation level and pasture management; and *εi* is the random residual error.

The average supplement intake of lambs was analyzed using ANOVA, adjusted for the covariate, according to the following model: Yc = µ + Ts + Tp + I + (Ts × Tp) + εi, where *Yc* is the observed intake value; *µ* is the overall mean; *Ts* is the effect of supplementation level; *Tp* is the effect of pasture management; *I* is the effect of the evaluation period (30, 60, and 90 days); *Ts × Tp* represents the interaction between supplementation level and pasture management; and *εi* is the random residual error.

Pasture intake estimates were analyzed by ANOVA, and means were compared using Tukey’s test at a significance level of 0.05. The statistical model used was: Ysp = µ + Ts + Tp + I + (Ts × Tp) + εi, where *Ysp* is the observed pasture intake value at 0–30, 30–60, and 60–90 days; *µ* is the overall mean; *Ts* is the effect of supplementation level; *Tp* is the effect of pasture management; *I* is the effect of evaluation period; *(Ts × Tp)* represents the interaction between supplementation level and pasture management; and *εi* is the random residual error.

The data related to EPG and anthelmintic treatments were analysed by ANOVA according to the following statistical model: Yi = µ + Ts + Tp+ I + B+ S×P + εi, where Yi is the observed value for the EPG variable; µ is the overall constant; Ts is the effect of supplementation level (0.5 or 1.0% BW); Tp is the effect of pasture management (mowed and non-mowed pasture); I is the effect of the sampling period (0,30,60, and 90 days postpartum); B is the covariate; SxP represents the interaction between supplementation level and pasture management; and εi is the random error associated with each observation. The initial EPG values of the sheep were included as a covariate in the statistical analysis to adjust the response variables.

The EPG data and anthelmintic treatment frequency were initially evaluated for dispersion, skewness, and kurtosis, followed by a normality assessment using the D’Agostino–Pearson test. EPG values were transformed using log 10 (x + 1) prior to analysis. The transformed EPG data and the absolute values of anthelmintic treatments were analyzed by ANOVA in a 2 × 2 factorial arrangements (supplementation level × pasture management). Mean comparisons were performed using Tukey’s test at a significance level of 0.05.

## Results

No interaction was observed between supplementation levels and pasture management on lamb productive performance or BCS. Likewise, ewe nutritional level and pasture management had no effect on lamb body weight or BCS at 30 and 90 days of age. The supplementation at 1.0% of ewe body weight positively affected lamb weight gain and improved BCS at 60 days of age (Table 3).

**Table 3:** Lamb’s productive performance, body condition score (BCS), supplement intake, and estimated leaf blade intake of lambs according to maternal supplementation (S), pasture management (P), experimental period (age), and the S × P interaction

	Supplementation		Pasture management		SEM	*P*-value		
	0.5%BW	1.0%BW	Mowed	Non-mowed		Supplementation	Pasture	S*P
Performance								
Birth Weight (kg)	3.8	3.9	3.95	3.7	0.195	0.6178	0.475	0.6833
BW at 30 days (kg)	12.8	14	13.8	13	0.632	0.1895	0.3356	0.1513
BW at 60 days (kg)	21.0^b^	23.5^a^	22.4	22.1	0.849	0.0439	0.7839	0.4409
BW at 90 days (kg)	27.8	30.8	29.3	29.2	1.104	0.0657	0.947	0.2867
ADG (g/day)	298	267	283	282	0.011	0.0522	0.9712	0.2597
TWG (kg)	24	26.8	25.4	25.5	0.99	0.0543	0.9688	0.2625
Body condition score								
BCS at 30 days	2.5	2.7	2.7	2.5	0.107	0.1318	0.507	0.3305
BCS at 60 days	2.4^b^	2.8^a^	2.7	2.5	0.101	0.0097	0.1801	0.8471
BCS at 90 days	2.2^b^	2.5^a^	2.5^a^	2.2^b^	0.098	0.0179	0.0257	0.7719
Supplement Intake (g/day)								
Average daily intake	355	338	352	340	0.051	0.8165	0.8734	0.7211
0-30 d	21	44	29	36	0.014	0.2758	0.7293	0.1764
30-60 d	288	288	268	308	0.027	0.9855	0.334	0.6616
60-90 d	629	562	567	624	0.049	0.3506	0.4291	0.6202
Leaf blade Intake								
Average daily intake	1723.6	1564.25	1683.3	1649.8	66.36	0.0997	0.6888	0.2929
0-30 d	1078.4	986.7	1066	999.1	48.67	0.1895	0.3356	0.1513
30-60 d	1615.9^b^	1807.7^a^	1724.6	1699.1	65.39	0.0439	0.7839	0.4409
60-90 d	2368.9	2141.8	2259.4	2251.3	85.04	0.0657	0.947	0.2867

Means followed by distinct lowercase letters in the rows differ from each other by Tukey's test (p<0.05); BW = body weight; ADI= average daily intake; ADG= Average daily gain; TWG= Total weight gain; ADI = Average daily intake; SEM= Standard error of the mean

Regarding supplement intake by lambs in the creep-feeding system, no interaction was detected between supplementation levels and pasture management, and supplement intake was not influenced by ewe supplementation level or pasture management (Table 3). Estimated leaf blades intake by lambs was not affected by the interaction between supplementation levels and pasture management, nor by pasture management alone. In contrast, ewe nutritional level significantly influenced lamb leaf intake. At 30 and 60 days of age, supplementation at 1.0% of ewe body weight resulted in the highest estimated leaf intake by lambs (Table 3).

Lamb age significantly affected the evaluated variables, with supplement intake and body weight increasing with advancing age, whereas BCS decreased (Table 4).

**Table 4:** Productive performance, body condition score (BCS), supplement intake, and estimated leaf blade intake of lambs according to age

	Age			SEM	*P*-value
	30	60	90		
Supplement intake (g/day)	32^c^	288^b^	595^a^	0.026	< 0.0001
Body weight (kg)	13.4^c^	22.2^b^	29.3^a^	0.630	< 0.0001
BCS	2.6^ab^	2.6^a^	2.4^b^	0.107	0.0367
Leaf blade intake (g/day)	1031.0^c^	171,278^b^	2259.0^a^	48.54	< 0.0001
EPG	17^b^	684^a^	599^a^	111.82	< 0.0001
Anthelminc in lambs (%)	0^b^	59.5^a^	61.10^a^	6.0354	< 0.0001

**Fig. 2:**
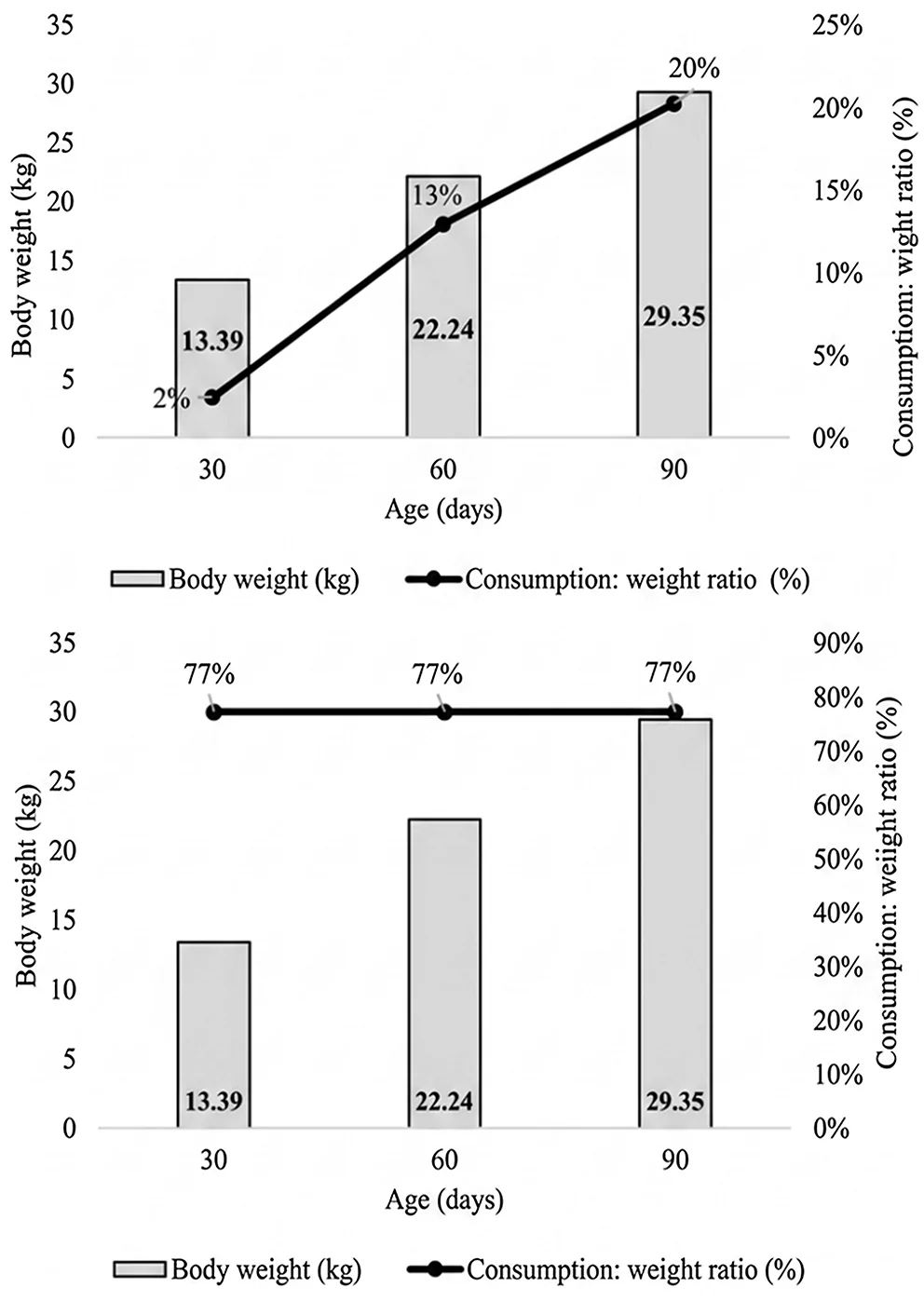
Relationship between supplement intake and pasture intake as a function of body weight of suckling lambs raised on Marandu grass pastures. Supplement intake = (body weight / supplement intake); Pasture intake = (body weight / pasture intake)

Supplement and pasture intake by lambs increased with age and body development. Supplement intake increased as a function of the intake-to-body weight ratio (Fig. 2). In contrast, pasture intake, expressed relative to the intake-to-body weight ratio, remained stable throughout the experimental period (Fig. 2).

An interaction between supplementation level and pasture management was observed for EPG in lambs. At 60 days of age, lambs born to ewes supplemented with 0.5% crude protein (on a body weight basis) and maintained on unmowed pastures exhibited higher EPG values than lambs from ewes receiving 1.0% BW supplementation under the same pasture management conditions. At 90 days of age, lambs grazing unmowed pastures and born to ewes supplemented with 1.0% BW showed higher EPG values compared with lambs grazing mowed pastures and born to ewes supplemented with 0.5% BW (Table 5).

**Table 5:** Mean eggs count per gram (EPG) at 30, 60, and 90 days of age and percentage of lambs requiring anthelmintic treatment during the 30–60 and 60–90 day intervals, according to ewe supplementation (S), pasture management (P), age, and the S × P interaction

	Treatment				SEM	P-value		
	0.5%BW		1.0%BW			Supplementation	Pasture	S*P
	MP	NMP	MP	NMP				
EPG 30 days	17	8	5	14	8.5337	0.5803	0.9118	0.1378
EPG 60 days	555^ab^	944^a^	586^ab^	547^b^	301.34	0.3954	0.4171	0.0399
EPG 90 days	205^b^	637^ab^	655^ab^	794^a^	176.68	0.0533	0.0686	0.0102
Lambs 30-60 days (%)^A^	69.23^b^	76.46^a^	53.85^c^	33.33^d^	0.4698	<0.0001	<0.0001	<0.0001
Lambs 60-90 days (%)^B^	30.77^d^	66.67^c^	76.92^b^	83.33^a^	0.5077	<0.0001	<0.0001	<0.0001

Means followed by different letters in the rows differ for interaction between supplementation and pasture management strategies by Tukey''''s test (P< 0.05); MP=Mowed pasture; NMP= Non-mowed pasture; SEM== Standard error of the mean

^A^Dewormed lambs (%) = (number of dewormed lambs / total number of lambs × 100); Lambs 30-60 days (%) = 69.23 (9/13*100); 76.46 (9/12*100); 53.85 (7/13*100); 33.33 (4/12*100)

^B^ Dewormed lambs (%) = (number of dewormed lambs / total number of lambs × 100); Lambs 60-90 days (%) = 30.77 (4/13*100); 66.67 (8/12*100); 76.92 (10/13*100); 83.33 (10/12*100)

Anthelmintic administration was also affected by the interaction between supplementation level and pasture management. At 30 and 60 days of age, a higher proportion of lambs required anthelmintic treatment when born to ewes supplemented with 0.5% BW and managed under unmowed pasture conditions. In contrast, between 60 and 90 days of age, a higher proportion of anthelmintic treatments was observed in lambs from ewes supplemented with 1.0% BW under the same pasture management conditions. In addition, both EPG counts and the proportion of lambs receiving anthelmintic treatment were influenced by the experimental period. Lambs at 30 days of age exhibited lower EPG values and a lower frequency of anthelmintic administration compared with lambs at 60 and 90 days of age (Table 5).

Pasture intake and BCS were not significantly correlated with body weight or body condition score. A significant negative correlation was observed between average daily intake and body condition score. In contrast, average daily intake showed positive correlations with EPG and pasture intake, which were of moderate and high magnitude, respectively (Table 6).

**Table 6:** Pearson correlation coefficient between the traits weight, average daily supplement intake (ADI), body condition score (BCS), eggs per gram of feces (EPG), pasture intake (PI), and body weight (BW) of lambs

	ADI	BCS	EPG	PI	BW
ADI		-0.17*	0.40*	0.82*	0.82*
BCS	-0.17*		-0.22*	0.11	0.13
EPG	0.40*	-0.22*		0.29*	0.29*
PI	0.82*	0.11	0.29*		1,00
BW	0.82*	0.13	0.29*	1.00*	

* = *p* < 0.05; ns = *p*>0.05

The chemical composition of leaf tissues was not affected by the interaction between supplementation level and pasture management. However, pasture management influenced the concentrations of NDF and ADF in the leaves, with higher concentrations observed in mowed pastures (Table 7). Likewise, the chemical composition of the stem was not influenced by the interaction between supplementation level and pasture management. An isolated effect of supplementation level was observed for EE concentration in the stem, with higher EE concentrations in pastures where sheep received supplementation at 1.0% BW compared with those supplemented at 0.5% BW. In addition, a higher DM concentration was observed in the stem of pastures where sheep received supplementation at 1.0% BW (Table 7).

**Table 7:** Chemical and morphological composition of Marandu grass pastures mowed pasture (MP) and non-mowed pasture (NMP) management according to ewe supplementation levels (0.5 and 1.0% of body weight)

	Supplementation		Pasture		SEM	*P*-value		
	0.5%BW	1.0%BW	MP	NMP		Supplementation (S)	Pasture (*P*)	S*P
Leaf blades								
Dry matter (g/kg)	483.32	474.93	472.67	485.93	4.4256	0.7937	0.6612	0.6117
Mineral matter (g/kg DM)	45.66	42.61	44.11	44.15	0.4812	0.3443	0.9906	0.8087
Crude Protein (g/kg DM	67.31	65.17	66.82	65.59	0.3699	0.4182	0.6205	0.6272
Ethereal extract (g/kg DM)	19.42	19.81	19.49	19.74	0.2626	0.8374	0.8942	0.8831
Neutral detergent fiber (g/kg DM)	690.54	695.79	716.88^a^	669.46^b^	2.4177	0.7602	0.0081	0.4925
Acid detergent fiber (g/kg DM)	402.71	380.05	424.88^a^	358.33^b^	3.2171	0.3343	0.0054	0.0847
Total digestible nutrients (g/kg DM)	550.96	535.79	534.28	552.47	0.7441	0.1621	0.0961	0.4595
Stem								
Dry matter (g/kg)	594.49	527.08	529.63	546.94	2.4082	0.5159	0.6152	0.9823
Mineral matter (g/kg DM)	28.61	26.56	26.49	28.68	0.1703	0.4018	0.3714	0.2782
Ethereal extract (g/kg DM)	7.5^b^	11.2^a^	8.8	9.9	0.0810	0.0028	0.3269	0.3698
Neutral detergent fiber (g/kg DM)	800.56	804.38	792.69	812.31	1.2404	0.8268	0.2727	0.7033
Acid detergent fiber (g/kg DM)	598.76	559.91	567.25	591.42	2.1327	0.2083	0.4297	0.0524
Total digestible nutrients (g/kg DM)	503.99	502.37	507.27	499.08	0.5174	0.8268	0.2727	0.7033
Morphological Composition								
Fresh forage mass (kg/ha NM)	14508.1	15016.3	15178.1	14346.3	1.4505	0.8062	0.6882	0.1406
Dry forage mass (kg/ha DM)	8936.9	9479.4	9219.4	9196.9	0.8466	0.6540	0.9851	0.2356
Leaf mass (kg/ha DM)	9367.0	9021.0	9466.0	8921.0	0.1814	0.8936	0.8333	0.3945
Leaf blade (%)	9.0	8.5	9.1	8.5	1.3006	0.7697	0.7597	0.7132
Stem (%)	30.6	30.1	35.4^a^	25.3^b^	1.5234	0.8113	< 0.0001	0.1842
Senescent material (%)	42.1	43.7	36.9^b^	48.8^a^	3.6339	0.7481	0.0283	0.3778
Inflorescence (%)	18.3	17.7	18.6	17.4	2.6835	0.8612	0.7500	0.7963

NM: Natural matter; Means followed by distinct lowercase letters in the rows differ from each other by Tukey’s test (< 0.05); SEM: Standard error of the mean

The masses of green, dry and leaf forage in the pastures were not affected by the interaction between supplementation level and pasture management, and no isolated effects of the treatments were detected. In contrast, the proportions of stem + sheath and senescent material were influenced by pasture management. Higher proportions of stem + sheath were observed in mowed pastures, whereas higher proportions of senescent material were recorded in unmowed pastures. No differences were detected in the proportions of leaf blade and inflorescence among the evaluated treatments (Table 7).

Stem crude protein concentration and the leaf: stem ratio were affected by the interaction between supplementation level and pasture management, as well as by the isolated effect of pasture management. Higher crude protein concentrations in the stem were observed in unmowed pastures compared with mowed pastures when sheep received supplementation at 1.0% BW. Under the same supplementation level, unmowed pastures also exhibited a higher leaf: stem ratio (Table 8). For the energy: protein ratio of leaf blades, interaction effects were observed between supplementation level and pasture management, in addition to isolated effects of sheep nutritional level and pasture management. The highest energy: protein ratios were observed in unmowed pastures, regardless of the level of supplementation provided to the sheep (Table 8).

**Table 8:** Crude protein of stem leaf:stem ratio and Energy:protein ratio of Marandu grass pastures mowed pasture (MP) and non-mowed pasture (NMP) management according to ewe supplementation levels (0.5 and 1.0% of body weight)

	Treatments				SEM	*P*-value		
	0.5%BW		1.0%BW			Supplementation (S)	Pasture (P)	S*P
	MP	NMP	MP	NMP				
Stem - crude protein (g/kg)	23.9^b^	25.5^ab^	22.7^b^	28.0^a^	0.0538	0.4371	0.0001	0.0209
Leaf:stem ratio (%)	0.3	0.38	0.3	0.43^a^	0.0035	0.075	<0.0001	0.075
Energy:protein ratio (%)	7.89^c^	8.49^a^	8.19^b^	8.35^a^	0.0185	0.0412	0.0001	0.0011

NM: Natural matter; Means followed by distinct lowercase letters in the rows differ from each other by Tukey's test (<0.05); SEM: Standard error of the mean

## Discussion

Supplementation of ewes at 1.0% of BW resulted in an increase of 2.5 kg in body weight and 0.4 units in BCS of lambs at 60 days of age. However, the greater body weight observed in lambs appears to be more closely associated with pasture intake than with maternal nutritional level per se. Between 30 and 60 days of age, lambs born to ewes supplemented at 1.0% BW exhibited higher pasture intake, which coincided with the greater body weight recorded at 60 days. The high-magnitude positive correlation between body weight and pasture intake supports this interpretation and indicates that variations in pasture composition may directly influence productive performance in sheep.

Despite the high correlation among the aforementioned variables, no negative effect on productive performance was observed in lambs managed under a grazing system with mowed pasture, even in the presence of elevated concentrations of NDF and ADF in the leaf blades. Pasture NDF concentrations remained above the range of 550–600 g/kg of DM, values commonly considered limiting to voluntary forage intake (Elejalde et al. 2012). Nevertheless, these elevated NDF levels did not impair lamb performance; on the contrary, progressive weight gain was observed throughout the 90-day evaluation period, resulting in a weaning weight of approximately 30 kg, which may contribute to a reduction in finishing time (Silva et al. 2022).

Supplement and pasture intake increased as lambs advanced in age. Greater consumption of solid feeds is expected as a consequence of increased body volume (Gurgel et al. 2023). However, supplement and pasture intake exhibited distinct patterns throughout the experimental period. Supplement intake increased progressively with lamb age and the associated increase in body volume. These findings indicate that the intake of protein–energy supplements in a creep-feeding system promotes gastrointestinal tract development, thereby enhancing productive performance by supporting the nutritional requirements of lambs during periods of rapid growth and weight gain (NRC 2007).

As lamb age and body volume increased, absolute pasture intake increased accordingly. However, when forage intake was expressed relative to body weight, no differences were observed throughout the evaluated period. The simultaneous provision of protein–energy supplementation and forage can be explained by the associative effects of supplementation on forage intake and dietary digestible energy (Moore et al. 1999). In the present study, an additive response was observed, whereby pasture intake remained unchanged with increasing supplementation levels, while total dry matter intake (pasture plus supplement) increased, resulting in greater weight gain in lambs. Comparable results were reported by Melo et al. (2017), who observed similar grazing times between lambs supplemented in a creep-feeding system and non-supplemented lambs.

This response can be attributed to the low nutritional value of *Urochloa* spp., characterized by reduced CP and total digestible nutrient concentrations, together with high NDF levels. These characteristics are commonly associated with reduced dry matter intake due to lower digesta passage rates and limited fiber degradation within the gastrointestinal tract (Moore et al. 1999). Consequently, forage intake remained unchanged despite increasing levels of supplementation, as voluntary pasture consumption under these conditions is primarily constrained by the intrinsic structural and chemical characteristics of the forage (Goes et al. 2005).

Supplementation of lactating ewes at 1.0% of body weight improved the BCS of their offspring at 60 and 90 days of age, whereas lambs managed on mowed pastures exhibited higher BCS at 90 days. The decline in BCS observed between 60 and 90 days coincided with an increase in EPG, associated with advancing age and greater exposure of lambs to the infective stages of gastrointestinal helminths. From approximately 60 days of age onwards, increased forage intake enhances the ingestion of infective larvae present on pasture, particularly in systems based on *Urochloa* spp., thereby favoring the establishment and intensification of gastrointestinal parasitism (Melo et al. 2017).

From a physiological perspective, gastrointestinal parasitism impairs the digestive and metabolic efficiency of lambs by increasing endogenous protein losses, reducing nutrient absorption, and reallocating energy and amino acids toward immune responses, thereby limiting growth and body tissue deposition (Coop and Kyriazakis 1999, 2001). These effects are exacerbated in young lambs due to their still-developing immune system, which enhances the negative impact of parasitic infections on productive performance and body condition. Although BCS did not exhibit a strong correlation with the other evaluated variables, the significant negative correlation—albeit of low magnitude—between BCS and EPG indicates that increased parasite burden contributed to reductions in body condition, particularly during periods of rapid growth and elevated nutritional demand.

Lambs maintained on unmowed pastures exhibited higher EPG at 60 days of age, irrespective of the nutritional level of the ewes. According to Arco et al. (2024), unmowed pastures are characterized by greater sward height, which promotes soil shading and the establishment of a microclimate with increased moisture and reduced solar radiation. These conditions enhance the survival, development, and availability of gastrointestinal helminth eggs and infective larvae on pasture. Consequently, this environment facilitates larval migration and persistence within the grazing stratum, thereby increasing the probability of ingestion by lambs, particularly as forage intake increases with advancing age (Roberto et al. 2023).

A moderate positive correlation was observed between pasture intake and EPG, indicating that higher parasitic burdens were associated with increased forage intake as a consequence of advancing age and greater body volume. From 60 days of age onwards, lambs showed a pronounced increase in pasture consumption, which likely intensified exposure to infective stages of gastrointestinal helminths present in the grazing environment, thereby contributing to the observed increase in EPG values. In the nutritional treatment with supplementation at 1.0% of BW, lambs grazing on unmowed pastures exhibited higher EPG values at 90 days of age. According to Gasparina et al. (2021), following substantial ingestion of infective larvae of *Haemonchus contortus*, the prepatent period required for egg excretion in the feces ranges from 15 to 21 days. This interval is critical for the expression of increased EPG values and likely explains the rise in parasitic burden observed in lambs during this phase of the experimental period.

Pasture intake increased with advancing age and body volume of the lambs, and a moderate positive correlation was observed between BW and EPG. This finding indicates that prolonged exposure to parasitic challenge, associated with increased forage intake, is related to a greater parasitic burden in the hosts. The moderate positive correlation between EPG and body weight suggests enhanced resilience of the lambs, as heavier animals were able to sustain satisfactory productive performance despite higher levels of parasitism. Moreover, the provision of 300 g of protein–energy supplementation in a creep-feeding system for lambs grazing Marandu grass pastures contributed to increased resilience to gastrointestinal parasitism. This response is likely associated with improved ruminal nitrogen availability, which, when combined with starch supply, enhances microbial protein synthesis and supports the nutritional and immunological status of the animals (Adejoro et al. 2020). Numerous studies have highlighted the role of protein–energy supplementation in enhancing resilience to gastrointestinal parasites in lambs managed on tropical pastures, resulting in improved productive performance under parasitic challenge (Melo et al. 2017; Roberto et al. 2023).

High concentrations NDF and ADF in the leaf blades of *Urochloa brizantha* cv. Marandu under mowed pasture management did not limit forage intake by lambs. Although elevated NDF concentrations are traditionally associated with reduced voluntary intake in ruminants due to physical fill constraints and decreased digesta passage rate (Van Soest 1994; Mertens 1997), the intake levels observed in the present study suggest a physiological adaptation of lambs to diets with a high proportion of structural carbohydrates. TDN concentrations remained stable throughout the experimental period and were not affected by the evaluated treatments, indicating a consistent dietary energy supply. This response may be attributed to enhanced chewing and rumination efficiency, adaptive shifts in ruminal microbiota, and increased fiber degradation, which collectively favor digesta passage rate and sustain intake even under high NDF conditions (Allen 2000; Poppi et al. 2000). These findings are in agreement with reports from tropical pasture systems during the dry season, which are typically characterized by high fiber concentrations and moderate energy availability (Melo et al. 2022; Arco et al. 2024).

The mechanical reduction of pasture height through mowing was intended to enhance soil exposure to solar radiation and wind, stimulate forage regrowth, and modify canopy structure, thereby increasing leaf blade availability. This management strategy directly affects ingestive behavior and the physiological mechanisms regulating intake in lambs, which are highly selective grazers with a marked preference for leaf blades. This morphological fraction is characterized by greater digestibility, lower concentrations of structural carbohydrates, and a higher ruminal passage rate, all of which favor increased voluntary intake (Van Soest 1994). In the present study, a higher leaf: stem ratio was observed in unmowed pastures, irrespective of the nutritional level provided to the ewes. Nevertheless, independent of pasture management and maternal supplementation, the proportion of leaf blades remained below the level considered adequate to meet the nutritional requirements of lambs during the suckling period (Silva et al. 2022).

This limitation can be attributed to the physiological and morphological characteristics of tropical pastures during the dry season, a period marked by reduced leaf elongation rates and increased tissue senescence, which result in greater accumulation of stems and dead material with high fiber concentrations and lower ruminal degradability (Gurgel et al. 2020). Under such conditions, voluntary intake is predominantly regulated by physical constraints, particularly ruminal fill and a reduced digesta passage rate, thereby limiting dry matter intake and the extent to which the nutritional requirements of growing lambs are met (Mertens 1997; Allen 2000). Consequently, the morphophysiological characteristics of *Urochloa brizantha* cv. Marandu pastures observed in the present study reinforce the relevance of protein–energy supplementation in creep-feeding systems as a strategy to enhance intake, improve digestive efficiency, and optimize the productive performance of lambs intended for meat production.

## Conclusion

The hypothesis that protein-energy supplementation at 1.0% of body weight for lactating ewes would improve the productive performance, body condition, and gastrointestinal parasitism of their lambs that were grazing on previously mowed pastures was not confirmed. The higher pasture intake observed in lambs born to ewes receiving 1.0% supplementation between 30 and 60 days of age was associated with increased body weight at 60 days. Advancing lamb age resulted in an absolute increase in pasture intake, concomitant with greater parasitic burden and body weight, indicating progressive exposure to parasitic challenge. Despite this, lambs exhibited increased tolerance and resistance to gastrointestinal parasitic infection when grazing *Urochloa brizantha* cv. Marandu. The provision of an average of 300 g/day of protein–energy supplementation to lambs under a creep-feeding system contributed to resilience to parasitism. Moreover, the high fiber concentrations and moderate total digestible nutrient levels in the leaf blades of mowed pastures did not restrict pasture intake, which remained constant when expressed relative to body weight. The progressive productive development of lambs throughout the 90 days resulted in weaning weights of approximately 30 kg, a level considered suitable for initiating the finishing phase and reducing the overall production cycle of lamb meat under tropical pasture conditions.

## Data Availability

The datasets generated and/or analyzed during the current study are available from the corresponding author in reasonable request.
